# Severity-Dependent Profile of the Metabolome in Hypospadias

**DOI:** 10.3389/fped.2020.00202

**Published:** 2020-04-24

**Authors:** Coriness Piñeyro-Ruiz, Nataliya E. Chorna, Marcos Raymond Pérez-Brayfield, Juan Carlos Jorge

**Affiliations:** ^1^Department of Anatomy and Neurobiology, School of Medicine, University of Puerto Rico, San Juan, United States; ^2^Department of Biochemistry, PR-INBRE Metabolomics Research Core, University of Puerto Rico, Medical Sciences Campus, San Juan, United States; ^3^Department of Surgery, Urology Section, School of Medicine, University of Puerto Rico, San Juan, United States

**Keywords:** hypospadias, severity, etiology, metabolites, metabolome, metabolomics

## Abstract

**Background & Objective:** Hypospadias, characterized by the displacement of the opening of the urethra at any point in the medial-ventral side of the penis, is classified upon severity as mild (Type I) and severe (Type II and Type III) hypospadias. Hypospadias' etiology is idiopathic in the majority of cases, and underlying causes seem of multifactorial origin. Studies regarding genetic variants support this notion. It is unknown whether downstream gene products fit this profile. This study evaluated the metabolome of hypospadias by using the emerging technology of metabolomics in the search for distinct cellular processes associated with hypospadias' etiology according to the severity of this congenital urogenital condition.

**Methods:** Foreskin samples were collected during urethroplasty from boys with Type I, II, and III hypospadias or undergoing elective circumcision (*N* = 28) between 5 and 28 months of age. Samples were processed and submitted to gas chromatography-mass spectrometry (GC/MS). MetaboloAnalyst (http://www.metaboanalyst.ca/) online platform was used for bioinformatic analyses.

**Results:** Thirty-five metabolites across experimental groups were identified by GC/MS. Principal component analysis (PCA) and partial least squares-discriminant analysis (PLS-DA) showed that the metabolome of Type II and Type III hypospadias patients differs from the metabolome of Type I hypospadias and control patients. Of those 35, 10 amino acids were found in significantly low concentrations in severe hypospadias: aspartate, glutamate, glycine, isoleucine, leucine, lysine, methionine, phenylalanine, proline, and tyrosine. A high concentration of the amino acid lysine was detected in mild hypospadias.

**Conclusions:** The observed downregulation of specific amino acids in severe hypospadias provides alternative routes for future research aiming to identify disrupted networks and pathways while considering the severity of hypospadias.

## Introduction

Hypospadias is characterized by the opening of the urethra at any point in the ventral side of the penis rather than at its usual position at the tip of the glans. It is classified depending upon severity as: mild (Type I), moderate (Type II), and severe (Type III) hypospadias. It is further grouped as mild (Type I) and severe (Type II & III) hypospadias (1, 2). Approximately 70% of cases are mild hypospadias, and 30% are severe cases (3–7). Given the marked differences in the prevalence of each subtype, a working hypothesis is that hypospadias' etiology may be severity-dependent. Moreover, molecular studies support this emergent scenario in hypospadiology.

Clinical studies assessing genetic variants (single nucleotide polymorphisms; SNPs) show differences between hypospadias' sub-types. Carmichael and colleagues (8) found an increased risk association for several diacylglycerol kinase kappa (*DGKK*) gene SNPs for mild to moderate hypospadias but not for severe hypospadias. In addition, significant associations of *DGKK* SNPs variants were found by Xie and colleagues (9) for mild and moderate hypospadias when compared to controls, but not for severe hypospadias when compared to controls. Another study found that the distribution frequency of CC/CT/TT genotypes in the ryanodine receptor 1 (*RYR1*) gene was significantly different between patients with mild hypospadias in comparison to patients with moderate to severe hypospadias (10). Differences in SNPs variants have been found in genes related to sex hormone biosynthesis and metabolism. SNPs in the hydroxysteroid (17-beta) dehydrogenase 3 (*HSD17B3*), hydroxy-delta-5-steroid dehydrogenase, 3 beta-and steroid delta-isomerase 1 (*HSD3B1*), and stAR-related lipid transfer domain containing 3 (*STARD3*) genes were associated to an increased hypospadias risk for moderate and severe hypospadias but not for mild hypospadias (11). Another study found a significant association of an SNP in the androgen receptor (*AR*) gene with an increased risk for severe hypospadias; this association was not found in mild cases of hypospadias (12). At the protein level, findings also differ between hypospadias severities. Protein expression level of c-Jun N-terminal kinase 2 (*JNK2*), involved in cell migration, was found significantly increased in penile skin tissue from boys with severe hypospadias in comparison to boys with mild hypospadias (13). In addition, *AR* protein expression was found at higher levels in preputial tissue from subjects with severe hypospadias than those with mild hypospadias and control subjects (14). Altogether, this evidence supports a severity-dependent etiology for hypospadias. However, it came to our attention that not many studies have assessed whether these severity-dependent differences are observed in downstream gene products.

The metabolome is the final downstream product of all cellular changes that occur at the genome, transcriptome, and proteome level (15–17). Metabolomics is a high throughput approach that provides the identification and quantification of the metabolome from a variety of sample types (18). Metabolomics is employed to assess congenital conditions such as congenital heart defects and congenital glaucoma (19, 20) in search of novel biological candidates underlying these conditions.

We studied the metabolome from foreskin samples obtained from boys undergoing elective circumcision and boys undergoing hypospadias I-, II-, or III- repair with the hypothesis that each group will express a distinct profile of the metabolome. The goal was to determine whether a metabolomics approach could provide a first level of analysis pointing at distinct and novel cellular processes associated with hypospadias.

## Materials and Methods

### Human Samples

This study was approved by the Institutional Review Board (IRB) under the Human Research Subjects Protection Office (HRSPO) at the University of Puerto Rico, Medical Sciences Campus. Parents of children undergoing urethroplasty and boys scheduled for elective circumcision were recruited at the HIMA San Pablo Hospital, Caguas, Puerto Rico. Hypospadias severity was assessed by Marcos Raymond Pérez-Brayfield, MD (pediatric urologist, Faculty in Surgery, Urology Section, School of Medicine, UPR). Informed consent was obtained from legal parents or guardians. Foreskin samples were collected from boys with Type I, II, & III hypospadias as well as control boys between the ages of 5–28 months of age. The sample was obtained from available inner foreskin, which is largely comprised of mucous membrane. Both lateral and dorsal inner foreskin were present in most samples. Boys with suggestive clinical changes of balanitis xerotica obliterans (*BXO*) were excluded from the study. Hypospadias was the only clinical presentation for patients in the hypospadias group.

**Figure 1:**
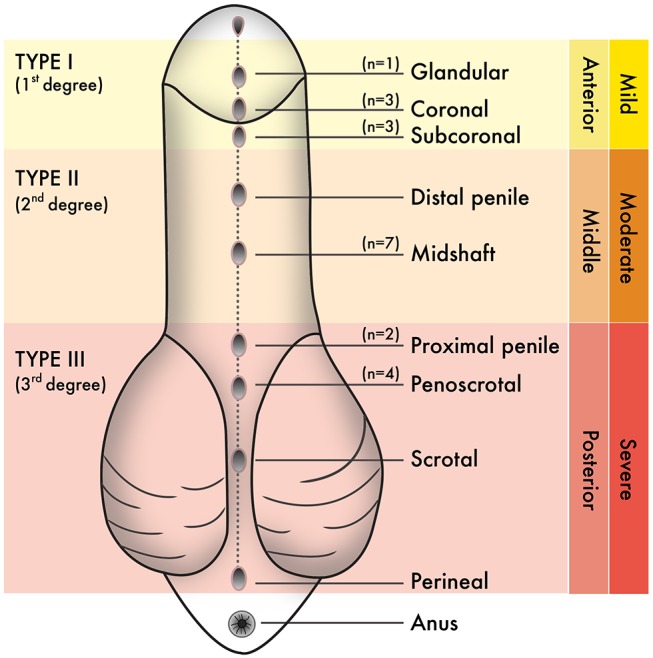
Staging or classification systems that describe the anatomical position of the urethral meatus in hypospadias have been created over the years. From these, “Type I–Type III” terminology is used in this study. *N* values correspond to the number of hypospadias cases reported hereafter penile degloving and chordee release during each urethroplasty procedure.

The population used in this study were boys born with hypospadias and/or undergoing elective circumcision in Puerto Rico. From June 2017 to June 2019, the estimated population of Puerto Rico within the time of sample collection was ~3 million people (21). The 2017 Annual Report of the Surveillance System of Congenital Defects from the Department of Health, Commonwealth of Puerto Rico, estimated that the prevalence of hypospadias in Puerto Rico was 24.86/10000 live births. Sample size estimation for our study included the following criteria: population size (for finite population correction factor or fpc) (N): 3000000; Hypothesized % frequency of outcome factor in the population (p): 0.25%+/−5; confidence limits as % of 100 (absolute +/– %)(d): 5%; Design effect (for cluster surveys-DEFF): 3. According to the Sample Size for Frequency in a Population formula (22) [sample size *n* = [DEFF^*^Np(1-p)]/[(d2/Z21-α/2^*^(N-1)+p^*^(1-p)], this study has an 80% confidence level with a total of 28 patients. These include seven patients with Type I hypospadias, seven patients with Type II hypospadias, six patients with Type III hypospadias, and eight patients undergoing circumcision as control subjects. The specific anatomical location of the urethral meatus was confirmed during the urethroplasty procedure after penile degloving by the pediatric urologist of the research team (MRP-B). Table 1 shows the average age of patients (in months) for each experimental group. Figure 1 shows the anatomical location of the urethral meatus for hypospadias patients that were included in this study according to various staging/classification systems. For reporting purposes, this study uses Type I-III terminology (1–2). Tissues were immediately frozen in dry ice and stored at −80°C until homogenization and processing for GC/MS.

**Table 1:** Average age (in months) at the time of urethroplasty.

**Groups**	**(Avg. ± SD)**
Controls	18.3 (9.11)
Type I	10.9 (7.56)
Type II	6.71 (1.98)
Type III	6.00 (0.926)

### Homogenization and Sample Preparation for Extraction of Metabolites

Fifty milligrams per sample were weighed and homogenized in 1 ml of chloroform/methanol/water (2:5:2), HPLC grade, by 15 s (3x) of sonication (on ice). Homogenates were shaken at 4°C for 15 min and centrifuged at 13,000 × 10 min at 4°C. The supernatant was completely evaporated. After evaporation, metabolites were derivatized by methoxyamination by adding 50 μl of 20 mg/ml solution of methoxyamine hydrochloride (Sigma-Aldrich, Catalog #: 226904) in pyridine (Sigma-Aldrich, Catalog #: 270407) and incubated at 37°C for 2 h. Trimethylsilylation was subsequently performed by adding 50 μl of N-methyl-N-trimethylsilyl-trifluoroacetamide (MSTFA+1% TMCS, Sigma-Aldrich; Catalog #: 375934) and incubated for 1 h at 65°C. Samples were centrifuged at 13,000 rpm for 10 min at RT. Supernatants were transferred to glass vials. Twenty microliters per sample were added to glass vials with inserts followed by the addition of 1 mM 2-Fluobiphenyl (Sigma-Aldrich, Catalog #: 102741) as an internal standard. Samples were processed by gas chromatography-mass spectrometry GC/MS-QP2010 (Shimadzu, Inc.) using analytical conditions as previously described (23).

### Data Processing and Bioinformatics

Raw chromatography data were obtained and processed in GCMS Solution Postrun Analysis software (Shimadzu) equipped with NIST14/2014/EPA/NIH database. After peak integrations for all metabolites and extensive mass spectral library searches of the major chromatographic peaks resulted in a final data set consisting of 35 metabolic features selected for the metabolomics analysis. Reproducibility of metabolite recovery, the performance of sample extraction, derivatization, and instrumentation were validated by the utilization of several blank samples, including a system suitability blank, extraction processing blank, and derivatization processing blank. To investigate the reproducibility of the metabolic features, a pooled composite sample was prepared from each experimental sample, aliquoted and processed similar to experimental samples as quality control (QC) (*n* = 3). To evaluate analytical accuracy and precision, an external quality assessment was performed using 2-Fluobiphenyl spiked into derivatization blank samples before running on the GC/MS (*n* = 6). The percent of relative standard deviation (%RSD) of 2-Fluobiphenyl peak abundances accounted for 7.3%, which demonstrates good reproducibility of the method. To mitigate systematic bias, we performed the randomization of the sample analysis order. Blanks and QC samples were spaced evenly among the injections to monitor instrument stability. Identified metabolites were transferred to a data matrix alongside retention time, peak area, and reference ions for each metabolite. Quantitative analysis of metabolic feature's concentrations in each sample was performed by calculation of a response factor using the internal standard. A table containing metabolite concentrations for each sample was uploaded in comma-delimited (^*^.csv) format to the MetaboAnalyst (24) online platform. MetaboAnalyst was used for data processing and statistical analyses that included data normalization, multivariate-, and univariate- analyses. Samples were normalized by sample weight and range scaling. Multivariate analyses consisted of principal component analysis (PCA) to assess variance between samples and partial least squares-discriminant analysis (PLS-DA) to identify separation between groups. Quality and reliability were assessed by cross-validation by using the parameters R2 and Q2, where R2 measures the degree of goodness in fit of the data, and Q2 measures quality assessment (17). PLS-DA significance was assessed by Permutation Test, where *p* ≤ 0.05 was considered statistically significant. Univariate analyses between Type I, II, & III hypospadias and control groups was assessed by one-way ANOVA with Fisher's LSD *post hoc* test. A *p* ≤ 0.05 was considered statistically significant. Significant values were submitted to Bonferroni correction (*p* < 0.05/14). PCA, PLS-DA, and Heatmap plots were generated in the MetaboAnalyst platform.

## Results

### Identification of Metabolites

**Figure 2:**
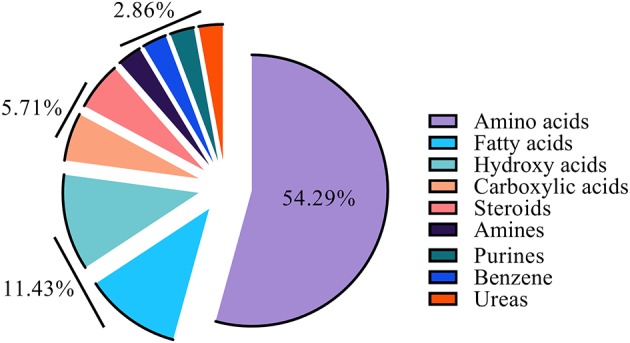
Constituent ratio of the 35 metabolites identified by class.

**Figure 3:**
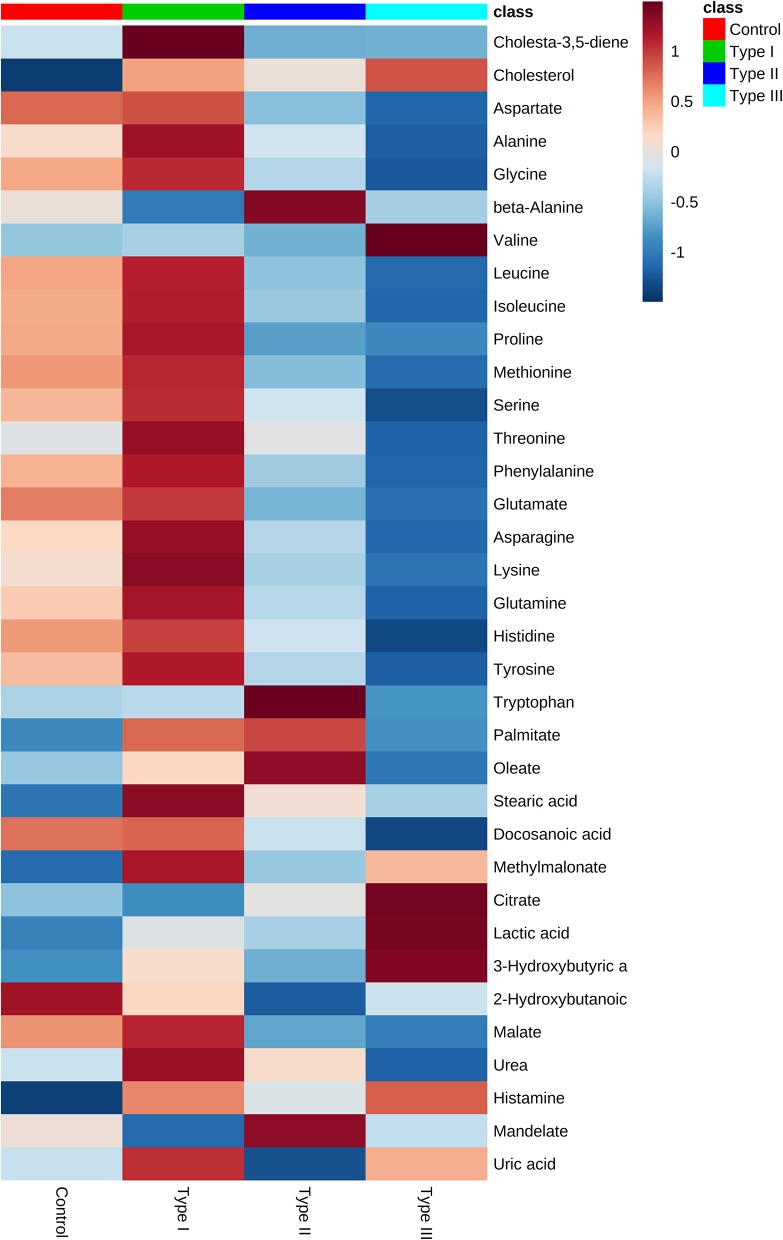
Metabolome of Type I, II, and III hypospadias, and controls. Heatmap displays average metabolites concentrations for each group. Control: *n* = 8; Type I hypospadias: *n* = 7; Type II hypospadias: *n* = 7; Type III hypospadias: *n* = 6. Blue represents low concentration; red represents high concentration.

Gas chromatography-mass spectrometry (GC-MS) was employed to identify and to quantify metabolites from foreskin samples as boys underwent elective circumcision or a urethroplasty procedure to manage hypospadias I-, II-, or III. A total of 35 metabolites were identified and classified as: amino acids (19); fatty acids (4), hydroxy acids (4), carboxylic acids (2), steroids (2), amines (1), purines (1), benzene (1), and urea (1) (Figure 2). Identified metabolites and their concentrations are appraised in Figure 3.

### Metabolic Differences Between Hypospadias Severities and Control Samples

**Figure 4:**
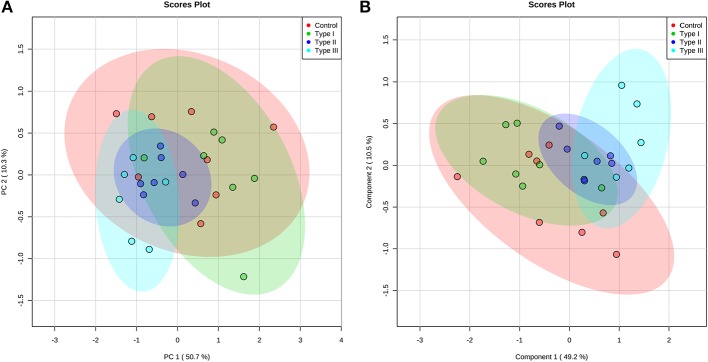
Type II and Type III hypospadias metabolome differs from Type I hypospadias and controls. **(A)** Principal component analysis (PCA) and **(B)** partial least squares-discriminant analysis (PLS-DA) analysis scores plots display variance and separation for Type II and Type III hypospadias between Type I hypospadias and control groups. The ellipses of the score plots illustrate 95% confidence region of the groups. Control: *n* = 8; Type I hypospadias: *n* = 7; Type II hypospadias: *n* = 7; Type III hypospadias: *n* = 6.

Multivariate analyses revealed differences in metabolic profiles between Type II and III hypospadias when compared to Type I hypospadias and control group. These metabolic differences were submitted to principal component analysis (PCA) to calculate variance between samples. PCA displayed a total of 61.0% variance, attributed to PC1 (50.7%) and PC2 (10.3%) components. Figure 4A depicts the 95% confidence ellipses for these groups. By using PCA score plots, we found that Type II and Type III hypospadias samples are more similar to each other and produce less variation than Type I hypospadias and control groups.

Furthermore, partial least squares-discriminant analysis (PLS-DA) displayed a total variance of 59.7%. PLS1 carried 49.2% of the total variance, whereas PLS2 contributed 10.5%. Type II and Type III hypospadias displayed satisfactory separation between Type I hypospadias and the control group, with a minor overlap of the 95% confidence ellipses (Figure 4B). On the contrary, Type I hypospadias and the control group showed no visible separation between them. PLS-DA was characterized by the following parameters: R2 = 0.359 and Q2 = 0.206. Permutation test revealed that separation between groups was statistically significant (*p* = 0.0265).

**Figure 5:**
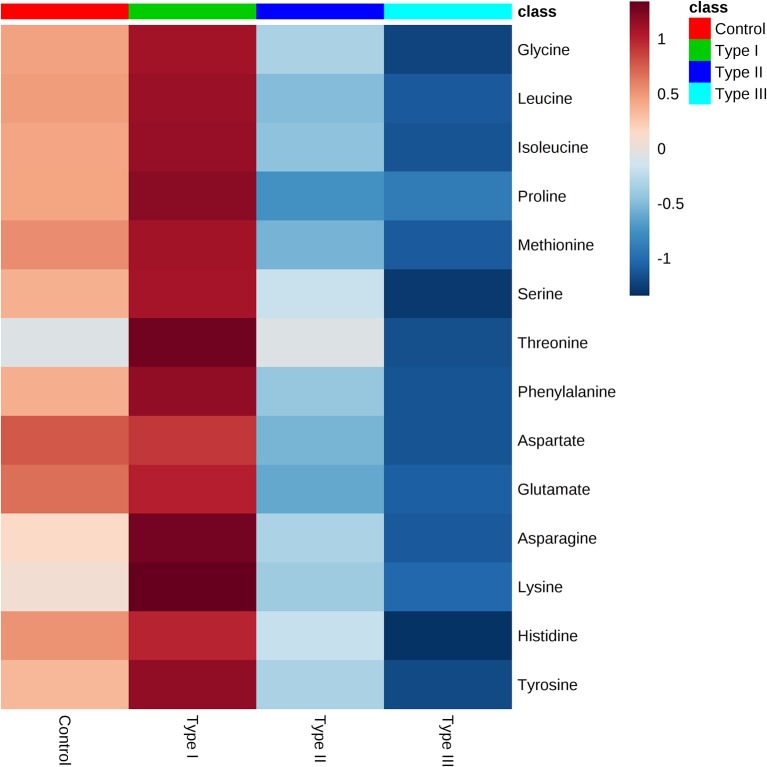
Significant downregulation of amino acids in Type II and Type III hypospadias. Heatmap represents metabolite concentrations (mM). Statistical analyses revealed that 14 amino acids had statistically significant lower concentrations in Type II and Type III hypospadias in comparison to Type I hypospadias and control groups. *p* < 0.05. Control: *n* = 8; Type I hypospadias: *n* = 7; Type II hypospadias: *n* = 7; Type III hypospadias: *n* = 6. Blue represents low concentration; red represents high concentration.

Univariate analysis was performed to evaluate whether there were statistically significant differences in metabolite concentrations between Type I, II, and III hypospadias and control groups. A one-way ANOVA followed by *post hoc* analysis revealed that 14 metabolites had statistically significant differences in metabolite concentrations between groups (Figure 5). After applying the Bonferroni correction, ten metabolites were identified: aspartate, glutamate, glycine, isoleucine, leucine, lysine, methionine, phenylalanine, proline, and tyrosine. Specifically, in comparison to the control group, Type I had one metabolite with higher concentrations, Type II had four metabolites with lower concentrations, and Type III had nine metabolites with lower concentrations. In comparison to Type I, ten metabolites were found with significantly lower concentrations in Type II and Type III hypospadias. These significant differences in metabolite concentrations between all experimental groups per metabolite are detailed in Table 2.

**Table 2:** Significant differences in metabolite concentrations between groups.

**Metabolite**	**Difference between groups**	***f*-value**	*p*-value^*^	**FDR**
Aspartate	Control vs. Type II & III; Type I vs. Type II & III	7.306	0.001	0.011
Glutamate	Control vs. Type II & III; Type I vs. Type II & III	6.370	0.002	0.013
Glycine	Control vs. Type III; Type I vs. Type II & III	5.677	0.004	0.015
Isoleucine	Control vs. Type III; Type I vs. Type II & III	8.074	0.001	0.009
Leucine	Control vs. Type II & III; Type I vs. Type II & III	8.113	0.001	0.009
Lysine	Control vs. Type I; Type I vs. Type II & III	5.833	0.004	0.015
Methionine	Control vs. Type III; Type I vs. Type II & III	6.152	0.003	0.013
Phenylalanine	Control vs. Type III; Type I vs. Type II & III	7.973	0.001	0.009
Proline	Control vs. Type II & III; Type I vs. Type II & III	6.325	0.003	0.013
Tyrosine	Control vs. Type III; Type I vs. Type II & III	7.004	0.002	0.011

* *Significant values after Bonferroni correction*.

## Discussion

To the best of our knowledge, this is the first study that assesses the metabolome of hypospadias severities by using human foreskin samples collected during urethroplasty. Our results demonstrate a tangible difference in the metabolome between mild and severe types of hypospadias, which further supports the notion based on previous genetics research work of plausible severity-dependent etiologies for hypospadias.

We were able to make a positive identification of 35 metabolites from inner foreskin samples through GC/MS analysis. The metabolome of Type II and Type III hypospadias displayed variance and separation from Type I hypospadias and control groups. Ten metabolites had significantly lower concentrations in Type II and Type III hypospadias in comparison to Type I hypospadias and control samples. These metabolites comprised the amino acids aspartate, glutamate, glycine, isoleucine, leucine, lysine, methionine, phenylalanine, proline, and tyrosine. While our study aimed to identify and quantify the metabolome of foreskin sample from hypospadiac boys, we speculate that the etiology of severe hypospadias might not just be the result of upstream dysregulations, such as genetic variants, protein synthesis or modifications, as it may be the result of reduced availability of downstream products that serve as metabolic intermediates and signaling molecules, such as amino acids. Amino acids have many functions, besides serving as structural units of proteins. Some of these functions include synthesis of hormones and neurotransmitters, serving as intermediates in signaling pathways, and as major metabolic intermediates for energy production such as in the citric acid cycle. Hence, our data supports the tenet that hypospadias' etiology is multifactorial, and that scarcity of amino acids may play an important role. In addition, although Type I hypospadias displayed an apparent upregulation of metabolites, only lysine had a significantly higher concentration from control samples. Therefore, the metabolomics profile in mild hypospadias is intriguing as it suggests a homeostatic mechanism to compensate for an altered developmental program. Further research is needed to address this hypothesis.

Aspartate is of particular interest as it was detected in lower concentrations in Type II and Type III hypospadias. Aspartate is a non-essential amino acid that is made from glutamine by enzymes using vitamin B6. This amino acid has essential roles in the urea cycle, DNA metabolism, and steroid hormone synthesis. In the rat model, it has been shown that aspartate induces the release of testosterone, luteinizing hormone, progesterone, and growth hormone release (25–28); entering the cell and increasing steroidogenesis in Leydig cells in the rat testis (26, 29). In humans, an oral dose of aspartate for 12 days increased testosterone and luteinizing hormone levels in serum in comparison to basal levels in men (30). Moreover, one study showed that aspartate upregulates the expression of androgen receptor protein levels in rat testis (28). We speculate that low concentrations of aspartate in Type II and III hypospadias might be related to circulating testosterone levels. This idea is consistent with previous work on genetic variants related to sex hormone biosynthesis and metabolism, as these have been associated with an increased risk for moderate and severe hypospadias but not for mild hypospadias (11). It is important to note that Type I hypospadias had similar aspartate concentrations to control samples. Therefore, from an embryological point of view, the etiology of Type I hypospadias may not be heavily influenced by androgen-dependent pathways.

Glutamate, isoleucine, leucine, phenylalanine, and proline are metabolites that are involved in energy metabolic processes. These metabolites were found in significantly lower concentrations in Type II and III hypospadias in comparison to Type I hypospadias and controls. Proline is a non-essential amino acid that is synthesized from glutamate. Glutamate is converted to alpha-ketoglutarate, one of the substrates involved in the rate-limiting step of the citric acid cycle. Phenylalanine, as well, is part of the citric acid cycle, as it is converted to acetoacetate and fumarate. Isoleucine and leucine are branched-chain amino acids which play critical roles in energy metabolism. These metabolites have been reported to participate in lipolysis, lipogenesis, glucose metabolism, among other energetic functions (31). Most importantly, branched-chain amino acids have been found to regulate mammary function and embryo development through the activation of the mechanistic target of rapamycin (*mTOR*) signaling pathway (31). The downregulation of these metabolites and their relationship to energy metabolic processes, including the citric acid cycle, lead us to speculate that the measured lower amino acid concentrations in severe hypospadias may be related to basic cellular processes that are essential for the differentiation of the male reproductive system.

Glutamate, methionine, glycine, isoleucine, leucine, lysine, and phenylalanine were found in significantly lower concentrations in severe hypospadias. These metabolites are associated with inborn errors of metabolism (32–38). Some examples of inborn errors of metabolism diseases are phenylketonuria, maple syrup urine disease, tyrosinemia type I, glycine encephalopathy, which involve the deficiency or accumulation of these amino acids (32). Moreover, Carmichael and colleagues (38) investigated whether hypospadias is associated with maternal dietary intake of nutrients related to one-carbon metabolism, including methionine. Data suggested that increased intake of methionine, choline, and vitamin B12 was associated with reduced risk of hypospadias. Thus, it deems vital that future studies investigate the plausible correlation between hypospadias risk and methionine intake as well as these newly identified metabolites.

There are a number of limitations of this study that warrants further research. First, aside from the atypical anatomical location of the urethral meatus, hypospadias is also characterized by atypical formation of the foreskin. Therefore, in search of hypospadias' etiology, the scientific rationale has been to study foreskin as a proxy measure of atypical location of the urethral meatus largely because it is absolutely unacceptable to collect urethral tissues for research purposes. The second one is related to the embryology of the urogenital systems, as it is not possible to research tissue samples of human urethral plates during embryonic and fetal development. Third, even though our study has an 80% confidence level, future studies with a larger sample size should confirm our findings. Fourth, analytical platforms for metabolomics other than the ones employed here could expand significant findings.

The age of the patient at the time of tissue collection and the type of tissue sample are inherent biases of this first study of the metabolome in hypospadias. Even though differences in age between experimental groups were dictated by best practices in the care of children born with hypospadias, this study assumes that such differences at the time of the urethroplasty procedure do not affect the metabolome. To further assess the reported differences seen here, it would be advantageous to submit to metabolomics analyses blood and/or urine samples along with inner foreskin samples from the same patient; both as an internal control and as an experimental strategy to address the question of whether age differences within infancy affect metabolomics profiles in hypospadias. In addition, this study employed inner foreskin tissue samples that would otherwise be discarded after the urethroplasty procedure. Available tissues, in most cases, included both lateral and dorsal inner foreskin tissues. Nevertheless, it is unknown whether specific histological features around the inner preputial coronal-shaft junction (39) differentially affect the metabolome in hypospadias. These inherent biases deserve further examination.

In conclusion, the metabolomics profile of Type II and Type III hypospadias patients differs from Type I hypospadias and control patients. The observed downregulation of specific amino acids in severe hypospadias provides alternative routes for future research aiming to identify disrupted networks and pathways while considering the severity of hypospadias. Our findings are consistent with the emergent working hypothesis in hypospadiology that favors the idea of severity-dependent etiologies underlying the atypical phenotypes of the male phallus at birth. Prenatal identification of these metabolites could lead to the development of biomarkers for hypospadias.

## Data Availability Statement

This data is available at the NIH Common Fund's National Metabolomics Data Repository (NMDR) website, the Metabolomics Workbench, https://www.metabolomicsworkbench.org where it has been assigned Project ID (PR000887). The data can be accessed directly via it's Project (doi: 10.21228/M81M59). This work is supported by NIH grant U2C-DK119886.

## Ethics Statement

This study was approved by the Institutional Review Board (IRB) under the Human Research Subjects Protection Office (HRSPO) at the University of Puerto Rico, Medical Sciences Campus. Written informed consent to participate in this study was provided by the participants' legal guardian/next of kin.

## Author Contributions

MP-B performed all surgical procedures and collected all tissue samples. CP-R assisted in sample collection and processed all tissue samples for metabolomics. CP-R conducted experiments under the guidance and expert advice of NC. NC evaluated the quality and reproducibility of data. JJ designed experimental protocol and cross-checked data for accuracy and supervised data analyses. MP-B and JJ provided advice during data collection. Depiction of data and writing of the first draft were by CP-R and JJ. All authors participated in the drafting and approval of the final version of the manuscript.

## Conflict of Interest

The authors declare that the research was conducted in the absence of any commercial or financial relationships that could be construed as a potential conflict of interest.
